# Interpretable AI for neural signal decoding in dementia: an EEG ensemble approach to differential diagnosis

**DOI:** 10.3389/fninf.2026.1902549

**Published:** 2026-08-27

**Authors:** Fawad Muhammad, Irfan Ahmed Usmani, Muhammad Aamir, Mai Alduailij, Mehrez Marzougui, Rab Nawaz

**Affiliations:** 1 Department of Biomedical Engineering, Salim Habib University, Karachi, Pakistan; 2 Department of Computer Sciences, College of Computer and Information Sciences, Princess Nourah Bint Abdulrahman University, Riyadh, Saudi Arabia; 3 College of Computer Science, King Khalid University, Abha, Saudi Arabia; 4 School of Computer Science and Electronic Engineering (CSEE), University of Essex, Colchester, United Kingdom

**Keywords:** electroencephalography (EEG), ensemble learning, explainable AI (XAI), machine learning, SHAP

## Abstract

**Introduction:**

The differential diagnosis between Alzheimer’s disease (AD) and frontotemporal dementia (FTD) presents a significant clinical challenge due to overlapping early-stage symptom profiles. Conventional resting-state EEG provides limited sensitivity to the impaired neural plasticity and lateralized cortical degeneration frequently observed in FTD.

**Methods:**

We developed a domain-informed heterogeneous ensemble framework incorporating dynamic neural reactivity and hemispheric asymmetry metrics from 19-channel EEG recordings acquired from 88 participants (36 AD, 23 FTD, 29 cognitively normal controls) during resting-state and photic stimulation paradigms. A neural reactivity vector (V_diff) was derived to quantify state-dependent spectral transitions. The 1,014-dimensional feature space was reduced to 200 features via recursive feature elimination, prioritizing spectral power distributions, hemispheric asymmetry indices (HAI), and stimulation-induced reactivity parameters. A weighted ensemble of Extreme Gradient Boosting (XGBoost) and Random Forest classifiers was evaluated using a subject-aware 90/10 holdout split with internal five-fold cross-validation.

**Results:**

The optimized model achieved a multi-class segment-level accuracy of 95.63% on an independently held-out test partition of 1,281 segments, with an internal five-fold cross-validation mean of 0.9846 ± 0.003. FTD-specific precision reached 0.9907. SHAP analysis identified beta-band hemispheric asymmetry and alpha-band reactivity as the principal contributors to class separation.

**Discussion:**

These findings indicate that the integration of dynamic state-transition measures with structural asymmetry proxies enhances electrophysiological discrimination between dementia subtypes. The framework provides a computationally efficient and biologically interpretable alternative to deep learning–based methodologies for EEG-driven dementia classification.

## 1 Introduction

Alzheimer’s disease (AD) and frontotemporal dementia (FTD) represent two of the most common neurodegenerative conditions worldwide, each characterized by distinct pathological signatures but overlapping early-stage clinical presentations. AD is typically associated with medial temporal lobe degeneration and progressive episodic memory impairment, whereas FTD predominantly affects frontal and anterior temporal regions, resulting in executive dysfunction and behavioral disturbances. Despite these differences, early phenotypic overlap frequently leads to diagnostic ambiguity and misclassification.

Given projections of a threefold increase in global dementia prevalence by 2050 (Nichols et al., 2022), improving subtype differentiation has become a critical clinical priority. Recent advancements have increasingly emphasized the development of non-invasive, AI-driven monitoring systems for Alzheimer’s and related dementias (Farhatullah et al., 2025). Accurate differentiation is critical, as reducing false-positive diagnoses can significantly mitigate the psychological trauma and pharmacological mismanagement associated with incorrect subtyping (White and Algeri, 2023). Electroencephalography (EEG) constitutes a non-invasive and economically accessible modality for the evaluation of functional brain dynamics (Umair et al., 2025). However, conventional resting-state EEG analyses primarily rely on static spectral power measures, which may inadequately capture impaired neural plasticity and thalamocortical responsiveness observed in FTD (Kang et al., 2025). Furthermore, lateralized cortical degeneration characteristic of FTD is not always fully reflected in global spectral metrics. These limitations suggest the need for physiologically informed feature representations capable of modeling both dynamic state transitions and structural asymmetry. Additionally, misdiagnosis can lead to contraindicated treatments, such as the use of acetylcholinesterase inhibitors in FTD patients, which may exacerbate behavioral symptoms (Perry and Hodges, 2000). In this study, we operationalize a reactivity-based framework by deriving a neural reactivity vector *V*_*diff*_, defined as the spectral power differential between resting-state and photic stimulation conditions. This measure aims to quantify state-dependent spectral transitions reflecting cortical responsiveness. In parallel, we introduce a Hemispheric Asymmetry Index (HAI) to model lateralized degeneration patterns by computing power differentials across homologous electrode pairs, serving as an electrophysiological proxy for structural asymmetry.

To integrate these domain-informed features, we implemented a heterogeneous ensemble framework combining Extreme Gradient Boosting (XGBoost) and Random Forest classifiers. Recursive feature elimination reduced the initial 1,014-dimensional feature space to 200 optimized predictors. Model interpretability was evaluated using SHapley Additive exPlanations (SHAP) analysis, ensuring that classification decisions were grounded in biologically meaningful biomarkers. Furthermore, for diagnostic AI to be clinically reliable, it must be grounded in neurophysiological reality rather than merely identifying statistical correlations within high-dimensional noise. Recent frameworks have demonstrated that integrating interpretable, multiview feature learning approaches to EEG data is essential for extracting reliable clinical biomarkers (Ahmad et al., 2025). Interpretability is therefore not merely a desirable feature but an increasingly considered important; decision logic must be transparent and traceable to established biological markers to ensure trust and safety (Freyer et al., 2024).

The primary contributions of this study are:

1. A novel formulation of dynamic neural reactivity features derived from photic stimulation, enabling the capture of state-dependent cortical responsiveness beyond conventional resting-state EEG analysis.

2. A physiologically grounded hemispheric asymmetry modeling framework (HAI), which operationalizes lateralized neurodegeneration patterns as discriminative biomarkers for frontotemporal dementia.

3. An interpretable and computationally efficient heterogeneous ensemble framework, demonstrating that high diagnostic precision in EEG-based dementia classification can be achieved without reliance on deep learning.

## 2 Methodology

### 2.1 Dataset

This study utilizes publicly available anonymized datasets and does not require institutional ethical approval. It complies with the Declaration of Helsinki. Two complementary open-access EEG datasets hosted on Open-Neuro have been utilized, provided by the 2nd Department of Neurology, AHEPA University Hospital of Thessaloniki.

Primary Dataset (ds004504): Curated by Miltiadous et al. (2021), this dataset contains resting-state eyes-closed (REST) recordings from a cohort of 88 participants, categorized into three groups: Alzheimer’s Disease (36 AD patients; mean age 66.4 ± 7.9), Frontotemporal Dementia (23 FTD patients; mean age 63.6 ± 8.2), and Cognitively Normal controls (29 CN subjects; mean age 67.9 ± 5.4) (Table 1). Recording durations averaged approximately 13.5 min for the AD group and 12 min for the FTD group (Miltiadous et al., 2021).

**TABLE 1:** Participant demographics and dataset structure.

Category	Alzheimer’s disease	Frontotemporal dementia	Cognitive normal	Total
Participants (n)	36	23	29	88
Mean age (SD)	66.4 (7.9)	63.6 (8.2)	67.9 (5.4)	
Recordings per subject	2 (REST, PHOTO)	2 (REST, PHOTO)	2 (REST, PHOTO)	176
Total sliding windows (4s)	5,593	3,365	3,953	12,911
Training samples (90%)	5,034	3,028	3,558	11,620
Test samples (10%)	559	337	395	1,281

Summary of cohort composition across Alzheimer’s disease (AD), frontotemporal dementia (FTD), and cognitively normal (CN) groups, including participant counts, mean age ( ± SD), recording structure, total segmented windows, and subject-independent train–test partition statistics.

Complementary Dataset (ds006036): Curated by Ntetska et al. (2025), this dataset features recordings from the same cohort of 88 participants including the 29 Cognitively Normal (CN) control subjects under eyes-open photic stimulation (PHOTO) conditions. It captures neural reactivity to multiple stroboscopic frequencies, with recordings lasting approximately 4.86 min for the AD group and 4.42 min for the FTD group. As both datasets were curated from the identical 88-subject cohort by the same research group, subject correspondence between REST and PHOTO recordings was established using the shared OpenNeuro participant identifiers, ensuring accurate subject-level pairing prior to computation of the DIFF (neural reactivity differential) (Ntetska et al., 2025).

The integration of the baseline resting-state (REST) and reactive photic-stimulation (PHOTO) datasets allowed for the isolation of neurologically relevant biomarkers while mitigating the influence of non-stationary artifacts. This multimodal approach is supported by research indicating that neurodegenerative impairment is often more visible during state-transitions than in isolation; specifically, a slowing of the neural response when shifting from baseline to photic stimulation serves as a sensitive indicator of impaired thalamocortical integrity and reduced neural plasticity (Yu et al., 2021). By capturing this dynamic reactivity through the DIFF vector, the framework prioritizes features with potential clinical relevance (Benz et al., 2014; Ryoo et al., 2025; Zheng et al., 2020). All EEG data were acquired using a 19-channel system (Fp1, Fp2, F7, F3, Fz, F4, F8, T3, C3, Cz, C4, T4, T5, P3, Pz, P4, T6, O1, and O2) at a sampling rate of 500 Hz. The raw data underwent a standardized automated pre-processing pipeline:

1. Spectral conditioning using a zero-phase Butterworth bandpass filter (0.5–45 Hz).
2. Re-referencing to linked mastoids to establish a neutral potential baseline.
3. Removal of non-stationary artifacts via the Artifact Subspace Reconstruction (ASR) routine.
4. Independent Component Analysis (ICA) followed by automated component rejection using the ICLabel routine in EEGLAB to eliminate biological interference (ocular and muscular artifacts).

### 2.2 Data processing and partitioning

Following preliminary preprocessing, the continuous EEG recordings were partitioned into non-overlapping 4-s sliding windows corresponding to 2,000 samples per segment. Windows with amplitudes exceeding 100 μV in any channel were automatically rejected to ensure signal purity, yielding a final dataset of 12,911 segments (REST: 6,455; PHOTO: 6,456).

To prevent identity leakage, all 12,911 segments were grouped by participant ID and a subject-aware 90/10 block partition was applied. This allocated 11,620 segments for training and internal validation, and 1,281 segments to a strictly isolated holdout test set (1,281 segments after artifact rejection). No participant contributed segments to both partitions. All final performance metrics including the 95.63% accuracy and the confusion matrix were computed at the segment level on this independent 10% holdout set.

### 2.3 Model architecture

Before training, features were scaled using a RobustScaler, which removes the median and scales according to the Interquartile Range (IQR) to provide resistance against EEG outliers. This technique is particularly effective for high-dimensional EEG feature spaces where non-stationary artifacts can introduce significant skewness; as demonstrated in recent work on EEG-based control systems (Angelakis et al., 2023), RobustScaler ensures that outliers do not disproportionately bias the learning objective, thereby improving the stability of downstream machine learning models. It is noted that the RobustScaler was fitted on the full 90% training partition prior to the internal five-fold cross-validation. The final 10% holdout test set was transformed using the pre-fitted scaler exclusively, ensuring zero leakage into the reported final evaluation metrics. The proposed diagnostic framework employs a heterogeneous ensemble architecture designed to balance bias and variance across the high-dimensional feature space. The classification core integrates two distinct algorithmic paradigms: Extreme Gradient Boosting (XGBoost) and Random Forest. The XGBoost component, configured with 1,500 estimators and a maximum depth of 12, serves as the primary learner. To address the prevalence of class imbalance inherent in neurodegenerative datasets, a custom cost-sensitive loss function was implemented, assigning a penalty weight of 2.0 to the minority Frontotemporal Dementia (FTD) class to encourage the model to learn minority-class spectral patterns. While the feature extraction phase generated a comprehensive vector of 1,014 dimensions, relying on such a high-dimensional space introduces significant risks of overfitting and reduces model generalizability (Liu and Gillies, 2016). To mitigate the Dimensionality issues, a Feature Optimization Protocol was applied prior to classifier training. A Random Forest-based Recursive Feature Elimination (RFE) strategy ranked features by their Gini impurity reduction (Menze et al., 2009). A systematic sweep analysis indicated that predictive performance stabilized at a subset size of *k* = 200 features. To rigorously prevent data leakage, the exact composition of this 200-feature subset was dynamically determined via RFE independently within the training split of each cross-validation fold. Consequently, the cross-validation accuracy reported in this study reflects the performance of this dynamic selection pipeline on entirely unseen test subjects. Following successful cross-validation, a final model was trained on the entire cohort (*N* = 88) using the same RFE protocol to extract the definitive 200-dimensional subspace used for subsequent SHAP interpretability analysis (Equation 1).


$$P_{f\,i\,n\,a\,l}=0.6\times P_{X\,G\,B\,O\,O\,S\,T}+0.4\times P_{R\,A\,N\,D\,O\,M\,F\,O\,R\,E\,S\,T}$$ (1)

The weighted soft-voting fusion is parameterized to prioritize the low-bias, high-precision decision boundaries of the boosting learner while utilizing the variance-stabilizing properties of the bagging component to prevent overfitting on the high-dimensional EEG feature space. This specific ratio is grounded in Stacked Generalization theory, which posits that combining learners with different inductive biases specifically the sequential error-correction of XGBoost and the parallel feature-subsampling of Random Forest jointly helps mitigate underfitting and overfitting than single-paradigm architectures (Opitz and Maclin, 1999). Effectively leveraging the granular decision boundaries of boosting while retaining the generalization of bagging.

**FIGURE 1:**
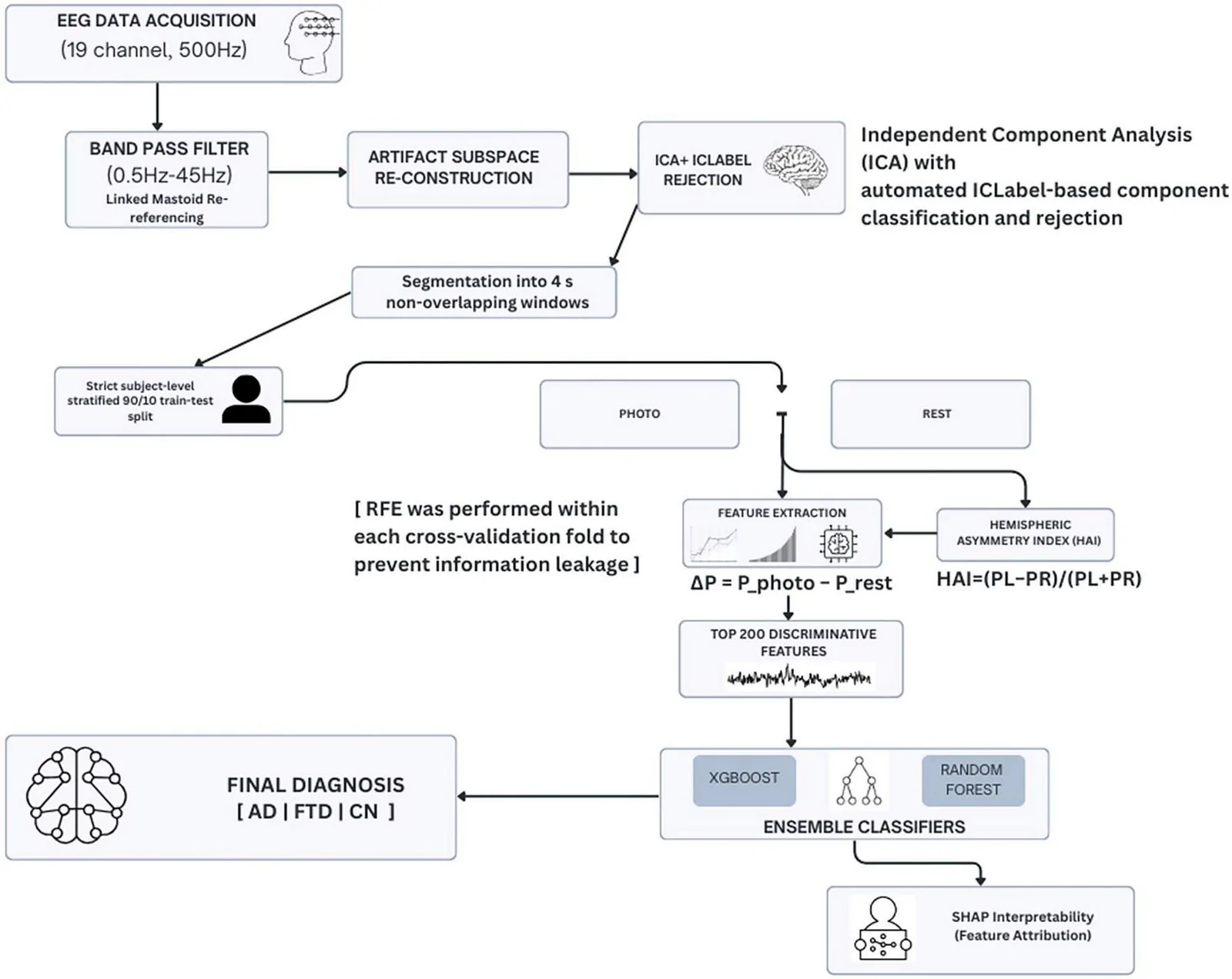
Schematic of the proposed EEG-based diagnostic framework. Automated preprocessing (bandpass filtering, ASR, and ICA) cleans the 19-channel EEG data before segmentation into 4-s windows. To strictly prevent data leakage, a subject-level 90/10 train-test split is applied prior to extracting multimodal features, including the Hemispheric Asymmetry Index (HAI) and dynamic reactivity. Recursive Feature Elimination (RFE) is executed exclusively within the training fold to select the top 200 discriminative features. These are fed into a weighted heterogeneous ensemble (XGBoost and Random Forest) for multi-class clinical diagnosis (AD, FTD, CN), with SHAP applied *post-hoc* for biological interpretability.

While 1,500 estimators represent high-capacity architecture, the use of parallel feature subsampling and conservative learning rates (0.02) served as critical regularizes, encouraging the model to prioritize global neurophysiological patterns. The proposed architecture is thus defined in Figure 1.

### 2.4 Hyperparameter configuration

Hyperparameters were selected via grid search within cross-validation.

- Ensemble size: Both the XGBoost and Random Forest models were configured with 1,500 estimators to ensure sufficient capacity for modeling the complex, high-dimensional feature space.
- Tree depth: A maximum depth of 12 was set for XGBoost to capture interaction effects without overfitting, while Random Forest utilized a depth of 30 to maximize the discriminative power of individual trees.
- Learning rate: The XGBoost learner utilized a conservative shrinkage rate of 0.02, ensuring gradual convergence and feature selection during the boosting process.
- Loss function: A custom weighted probabilistic loss was implemented to handle class imbalance, assigning a penalty weight of 2.0 to the minority FTD class and 1.0 to AD/CN classes.
- Fusion logic: Final predictions were generated via Weighted Soft-Voting, assigning a 0.6 coefficient to XGBoost and 0.4 to Random Forest to balance boosting precision with bagging stability. This computational rationale is grounded in the principle that heterogeneous ensembles can jointly overcome the limitations of model overfitting and underfitting by leveraging the high-precision decision boundaries of gradient boosting (low bias) while simultaneously mitigating variance through the stabilizing properties of bootstrap aggregation. This weighting specifically prioritizes the error-reduction capabilities of the boosting learner while retaining the strong generalization of the bagging component, which is critical for maintaining performance on small, high-dimensional clinical datasets (Johnson et al., 2022).
- Validation strategy: A subject-aware 90/10 holdout split was employed, with internal five-fold cross-validation applied exclusively within the training partition to ensure stability across diverse participant subsets (White and Power, 2023).
- Regularization: Random Forest employed bootstrap aggregation (bagging) to reduce variance, while XGBoost utilized shallow tree constraints to prevent the memorization of noise.

### 2.5 Feature extraction

Power spectral density (PSD) was computed using Welch’s method, employing a 256-sample window (approximately 0.512 s) with 50% overlap (128 samples), resulting in a frequency resolution of approximately 1.95 Hz. Features were extracted for the REST and PHOTO conditions, and a reactivity vector (DIFF = PHOTO-REST) was computed. This approach is grounded in the principle that dynamic connectivity markers capture temporal fluctuations and reactive states that static measures often average out, offering a more sensitive index of neural plasticity than isolated baseline recordings (Zheng et al., 2020).

Feature Optimization: The initial extraction phase yielded a comprehensive pool of 1,014 candidate features. To promote statistical parsimony and mitigate the risk of overfitting, the feature optimization protocol reduced the original feature space to the 200 most discriminative dimensions. The final model relies exclusively on this optimized subset. The Technical specifications of the EEG pre-processing and the feature optimization are summarized in Table 2.

**TABLE 2:** Technical specifications of EEG preprocessing and feature optimization.

Parameter	Specifications
Sampling rate	500 Hz
Segment duration	4.0 s (2,000 samples)
Spectral modalities	REST, PHOTO, DIFF (Reactivity)
Electrode system	19-channel international 10–20
Frequency resolution	∼1.95 Hz
Initial feature pool	1,014 dimensions
Final optimized vector	200 dimensions (selected via RFE)

Overview of signal acquisition settings, segmentation parameters, spectral feature modalities, electrode configuration, and dimensionality reduction workflow from the original high-dimensional feature space to the final optimized feature vector.

#### 2.5.1 Spectral and sub-band features

For each of the 19 channels, mean power was calculated for five standard clinical bands: Delta (0.5–4 Hz), Theta (4–8 Hz), Alpha (8–13 Hz), Beta (13–30 Hz), and Gamma (30–45 Hz). To capture FTD-specific signatures, we further extracted power from six fine-grained sub-bands:

- Low theta (4–6 Hz) and high theta (6–8 Hz)
- Low alpha (8–10 Hz) and high alpha (10–13 Hz)
- Low beta (13–20 Hz) and high beta (20–30 Hz)

Power values were log_10_ transformed to normalize the distribution.

**FIGURE 2:**
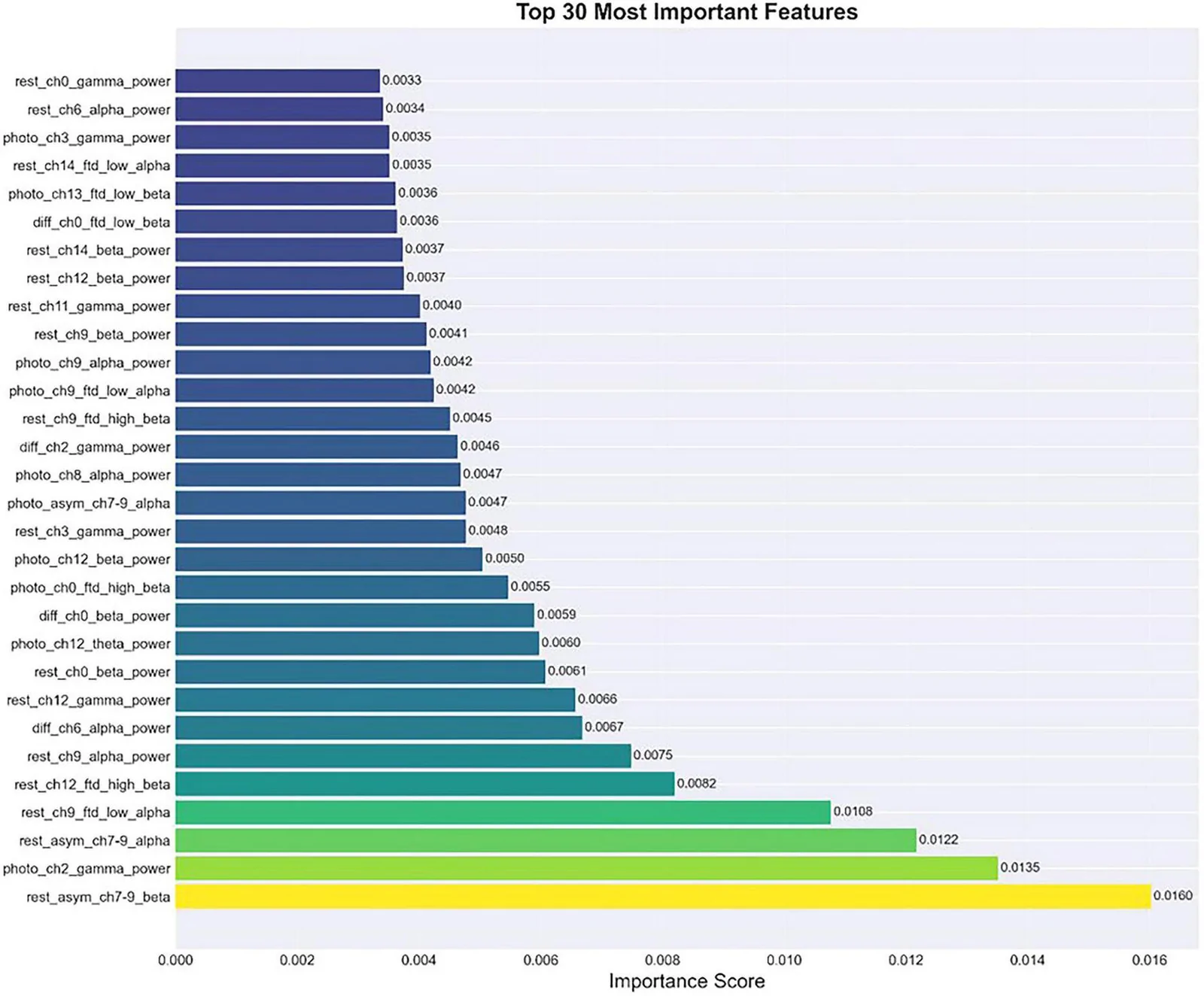
Top 30 discriminative features ranked by model importance (Gain). Extracted from the final ensemble model, the Resting-State Beta Asymmetry Index (rest_asym_ch7-9_beta) emerges as the strongest predictor, suggesting the diagnostic value of lateralized spectral power in FTD. The high ranking of dynamic markers like photic gamma power (photo_ch2_gamma_power) further helps the integration of reactivity metrics alongside standard resting-state topography.

The top 200 discriminative features are listed in Figure 2 with their Importance scores.

#### 2.5.2 Statistical and global biomarkers

For each channel, we computed five statistical descriptors: Mean, Standard Deviation, Skewness, Kurtosis, and Peak-to-Peak (PTP) amplitude. Additionally, global whole-brain features were integrated:

- Global relative power for each standard band.
- Alpha/Theta ratio.
- FTD-specific ratios (Low/High Theta and Low/High Alpha).
- Frontal Slowing Index (FSI): The ratio of Theta to Alpha power across frontal channels (Fp1, Fp2, F7, F3, Fz, F4, F8).

#### 2.5.3 Hemispheric asymmetry index (HAI)

To detect lateralized atrophy common in FTD, we calculated asymmetrical indices for seven homologous electrode pairs: Fp1-Fp2, F7-F8, F3-F4, T3-T4, C3-C4, P3-P4, and O1-O2. This ensures the model captures potential hemispheric power imbalances across all standard frequency bands, particularly in the temporal regions where neurodegeneration is most pronounced in FTD cohorts (Equation 2).


$$\frac{P_{l\,e\,f\,t}-P_{r\,i\,g\,h\,t}}{P_{l\,e\,f\,t}+P_{r\,i\,g\,h\,t}+10^{-10}}$$ (2)

The spectral and sub-band features (section 2.5.1) combined with the statistical and global biomarkers (section 2.5.2) and hemispheric asymmetry indices (section 2.5.3) yield 338 distinct features per condition. Applied across three conditions (REST, PHOTO, and DIFF = PHOTO - REST), this produces a final integrated feature vector of 1,014 dimensions (338 × 3) per segment. The inclusion of the Hemispheric Asymmetry Index (HAI) is motivated by structural MRI findings indicating that Frontotemporal Dementia (FTD) typically exhibits significantly more pronounced asymmetric atrophy patterns compared to Alzheimer’s Disease (AD), a phenomenon recently quantified via the Cortical Asymmetry Index (CAI) (Pérez-Millan et al., 2025). Nonlinear and fractal dynamical features, including Higuchi fractal dimension and Lyapunov exponents, have similarly been shown to capture disease-specific neural disruptions in EEG-based dementia and neurodegenerative disorder classification (Bunterngchit et al., 2024).

### 2.6 Performance evaluation metrics and model interpretability

Model evaluation was conducted using a combination of global and class-specific performance measures to ensure a rigorous and unbiased assessment. Specifically, accuracy, macro-average F1-score, Cohen’s Kappa coefficient, and class-wise metrics were computed. These metrics collectively capture overall predictive capability, per-class behavior, and agreement reliability beyond chance.

#### 2.6.1 Accuracy

Accuracy represents the proportion of correctly classified instances relative to the total number of observations (Equation 3):


$$\text{Accuracy}=\frac{T\,P+T\,N}{T\,P+T\,N+F\,P+F\,N}$$ (3)

#### 2.6.2 Precision

Precision reflects the proportion of instances predicted as positive that are correctly classified (Equation 4):


$$\text{Precision}=\frac{T\,P}{T\,P+F\,P}$$ (4)

#### 2.6.3 Recall (sensitivity)

Recall evaluates the ability of the classifier to correctly detect positive instances (Equation 5):


$$\text{Recall}=\frac{T\,P}{T\,P+F\,N}$$ (5)

#### 2.6.4 Specificity

Specificity reflects how effectively negative instances are identified (Equation 6):


$$\text{Specificity}=\frac{T\,N}{T\,N+F\,P}$$ (6)

#### 2.6.5 F1-score

The F1-score is defined as the harmonic mean of precision and recall, providing a balanced measure of classification performance (Equation 7):


$$\mathrm{F1}=\frac{2\,T\,P}{2\,T\,P+F\,P+F\,N}$$ (7)

#### 2.6.6 Macro-averaged F1-score

To ensure equal importance across all classes, the macro-averaged F1-score was computed as (Equation 8):


$$\mathrm{Macro}-\mathrm{F1}=\frac{1}{C}\,\overset{C}{\underset{i=1}{\sum }}F\,1_{i}$$ (8)

where **C** denotes the total number of classes and *F1_i_*is the F1-score of class *i*.

#### 2.6.7 Cohen’s kappa coefficient

Cohen’s Kappa quantifies the level of agreement between predicted and true class labels while adjusting for agreement expected by chance: $\kappa =\frac{p_{o}-p_{e}}{1-p_{e}}$

where: *p*_*o*_ = *observedagreementp*_*e*_ = *expectedagreementbychance* For binary classification (Equation 9):


$$p_{e}=\frac{\left(T\,P+F\,P\right)\,\left(T\,P+F\,N\right)+\left(F\,N+T\,N\right)\,\left(F\,P+T\,N\right)}{{\left(T\,P+T\,P+T\,N+F\,P+F\,N\right)}^{2}}$$ (9)

#### 2.6.8 Model Interpretability

To ensure clinical and physiological relevance, model interpretability was examined using SHAP (SHapley Additive exPlanations) and gain-based feature importance scores.

SHAP values quantify the marginal contribution of each feature to a prediction based on cooperative game theory (Equation 10):


$$\phi_{i}=\underset{S\subseteq F\backslash \left\{i\right\}}{\sum }\frac{\left|S\right|!\,\left(\left|F\right|-\left|S\right|-1\right)!}{\left|F\right|!}\,\left[f\,\left(S\cup \left\{i\right\}\right)-f\,\left(S\right)\right]$$ (10)

where:

*F* set of all features

*S* subset of features

*f*(*S*) model prediction using subset *S*

ϕ_*i*_ contribution of feature *i*

Gain-based importance, commonly used in tree-based models, measures the total reduction in impurity contributed by a feature across all splits (Equation 11):


$${\text{Gain}}_{j}=\Sigma \,\mathrm{Impurity}\,\mathrm{Reduction}\,\mathrm{from}\,\mathrm{splits}\,\mathrm{on}\,\mathrm{feature}\,j$$ (11)

These interpretability techniques enabled quantitative attribution of feature influence and verification that decision patterns learned were consistent with established neurophysiological markers of dementia. It is important to note that Gini-based gain importance, used here for RFE feature ranking, measures aggregate impurity reduction across tree splits; it is a training-data property. SHAP values, by contrast, quantify each feature’s marginal contribution to individual predictions via coalition averaging, making them a distinct and complementary attribution method.

### 2.7 Implementation environment and workflow

All experimental procedures were conducted on a dedicated computing workstation featuring an Intel^®^ Core i5 processor (6 cores, 12 threads), 16 GB of system memory, and an NVIDIA^®^ GeForce^®^ GTX 1660 Super graphics processing unit (GPU).

### 2.8 Statistical validation

To tune hyperparameters and assess internal stability, stratified five-fold Cross-Validation was applied exclusively within the 90% training partition (11,620 segments). In each fold, the RFE feature selection was recalculated independently using only the training subjects to prevent leakage into the validation fold. The mean cross-validation accuracy of 0.9846 ± 0.003 reflects segment-level performance across these internal folds. Statistical significance of the final predictions on the holdout set was assessed via Cohen’s Kappa coefficient, confirming agreement between predicted and true labels substantially exceeded chance. Ninety-five percent confidence intervals for the final evaluation were computed using the normal approximation method for a binomial proportion based on the segment-level predictions of the holdout test set.

## 3 Results

### 3.1 Classification performance

The heterogeneous ensemble achieved a segment-level accuracy of 95.63% on the independent 10% holdout test set (1,281 segments). Majority voting was applied across all segments belonging to each participant to illustrate subtype-level prediction consistency. Table 3 summarizes the performance of the proposed domain-guided heterogeneous ensemble approach.

**TABLE 3:** Diagnostic performance of the proposed domain-guided heterogeneous ensemble approach across Alzheimer’s disease, frontotemporal dementia, and cognitively normal groups, reported in terms of accuracy, precision, recall, and F1-score.

Diagnostic class	Accuracy	Precision	Recall (Sens.)	F1-score
Alzheimer’s (AD)	97.0%	0.919	0.970	0.943
Frontotemporal (FTD)	95.1%	0.9907	0.951	0.971
Cognitive normal (CN)	94.0%	0.930	0.940	0.935

**FIGURE 3:**
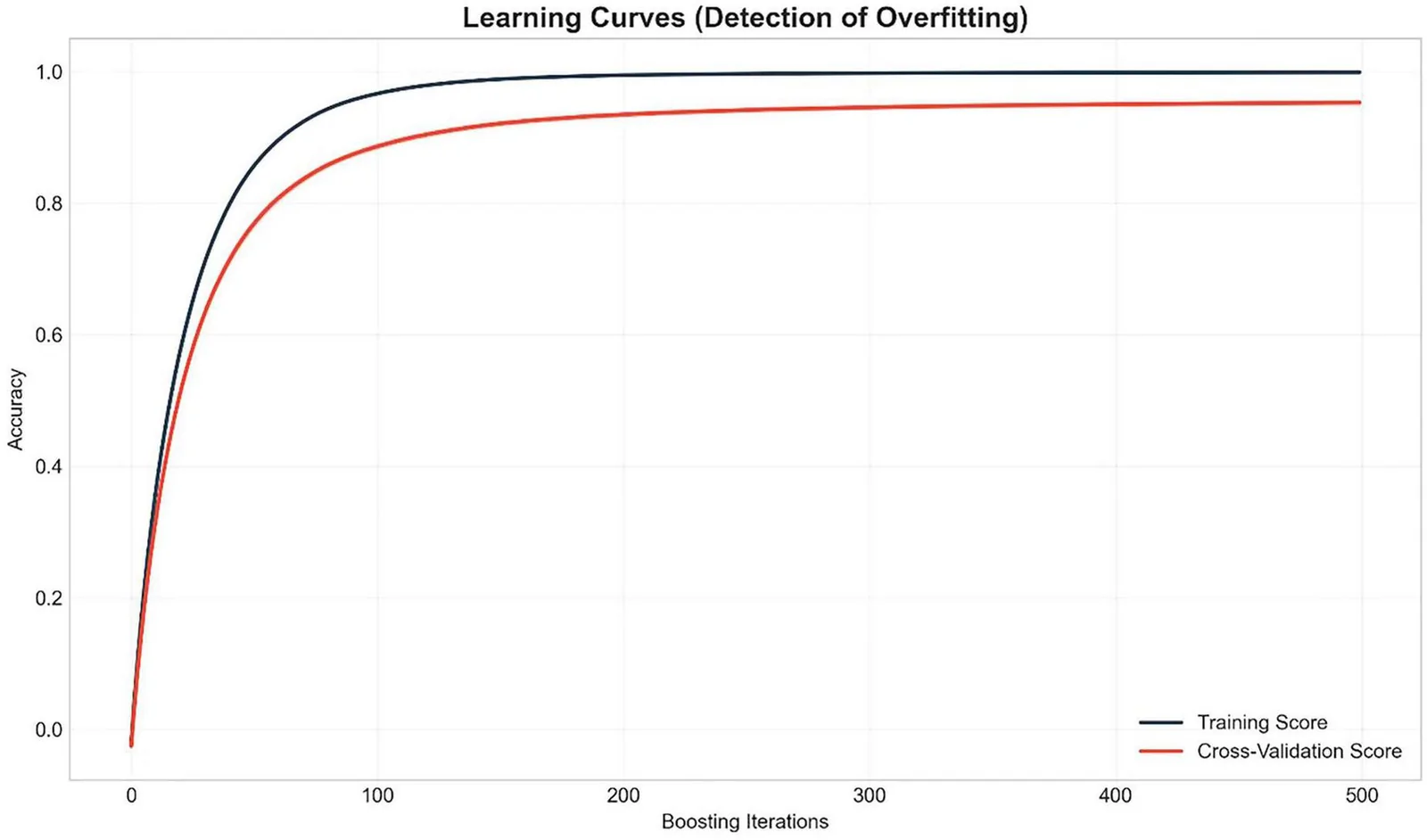
Learning curves for the boosting classifier. Training and cross-validation accuracy are plotted across 500 boosting iterations. The convergence of the validation score alongside the training trajectory confirms effective regularization and the absence of high-variance overfitting in the reduced feature space.

The framework demonstrated high sensitivity for the Alzheimer’s (AD) cohort (97.0%). Critically, the cost-sensitive learning objective was associated with improved performance for the Frontotemporal Dementia (FTD) minority class, achieving a precision of 0.9907. This high precision metric is noteworthy from a diagnostic perspective, as it reflects the model’s ability to minimize false-positive FTD diagnoses. As avoiding false positives is just as critical as minimizing false negatives, since it can results in significant psychological, medical, and financial consequences for the individual (White and Algeri, 2023). Figure 3 shows the Learning curves for the boosting classifier. Training and cross-validation accuracy are plotted across 500 boosting iterations.

### 3.2 Diagnostic discriminative analysis

The confusion matrix analysis of the holdout test set (1,281 segments) reveals distinct separation between neurodegenerative subtypes. The model correctly identified 428 out of 450 segment-level FTD instances, misclassifying only 4 as Cognitively Normal (CN). The minor classification errors observed primarily involved subtle overlaps between early stage FTD and AD spectral signatures, reflecting the known clinical difficulty in differentiating these pathologies solely via electrophysiological markers (Mlinarič et al., 2026).

**FIGURE 4:**
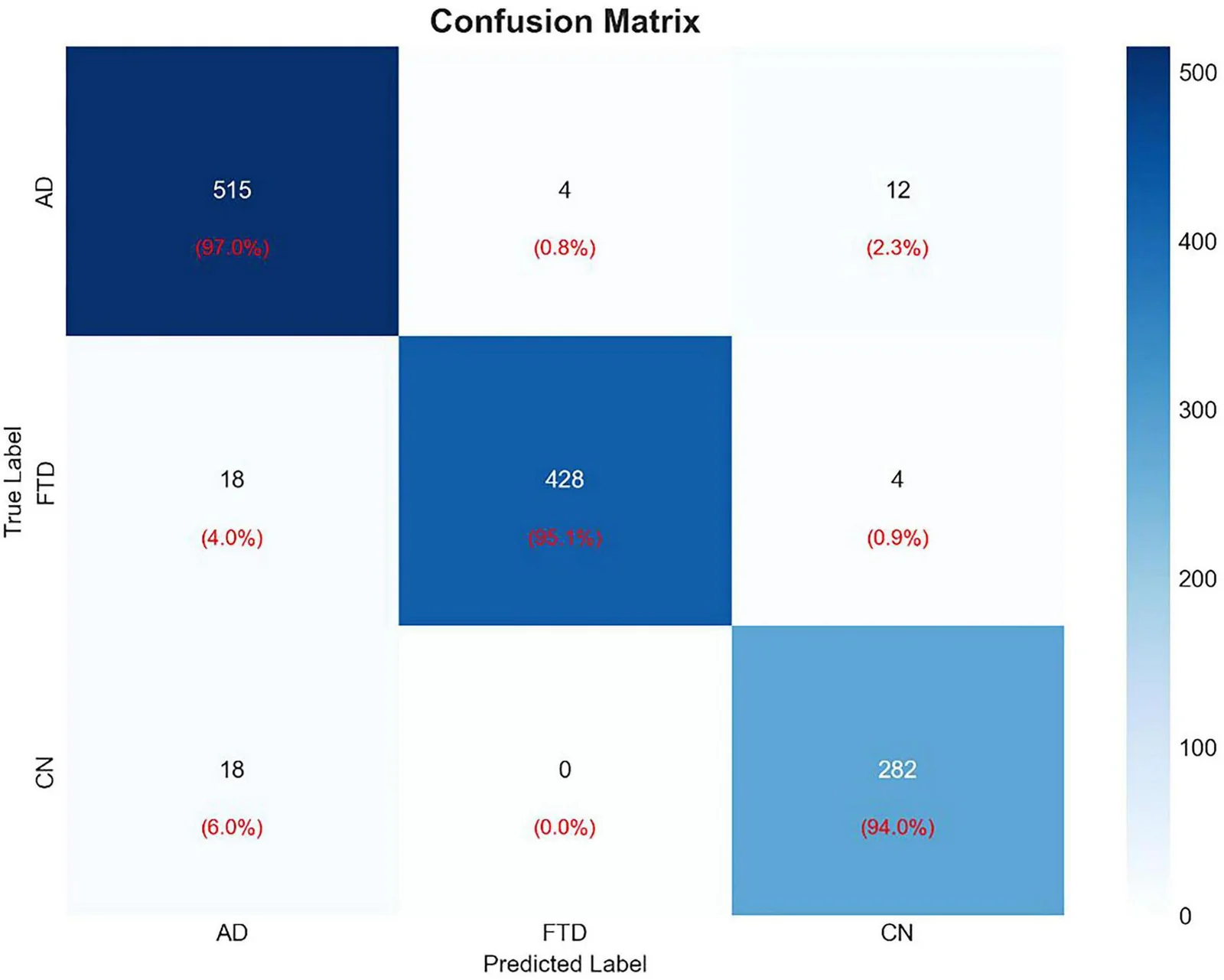
Subject-level confusion matrix. Rows denote true clinical labels and columns represent ensemble predictions.

**FIGURE 5:**
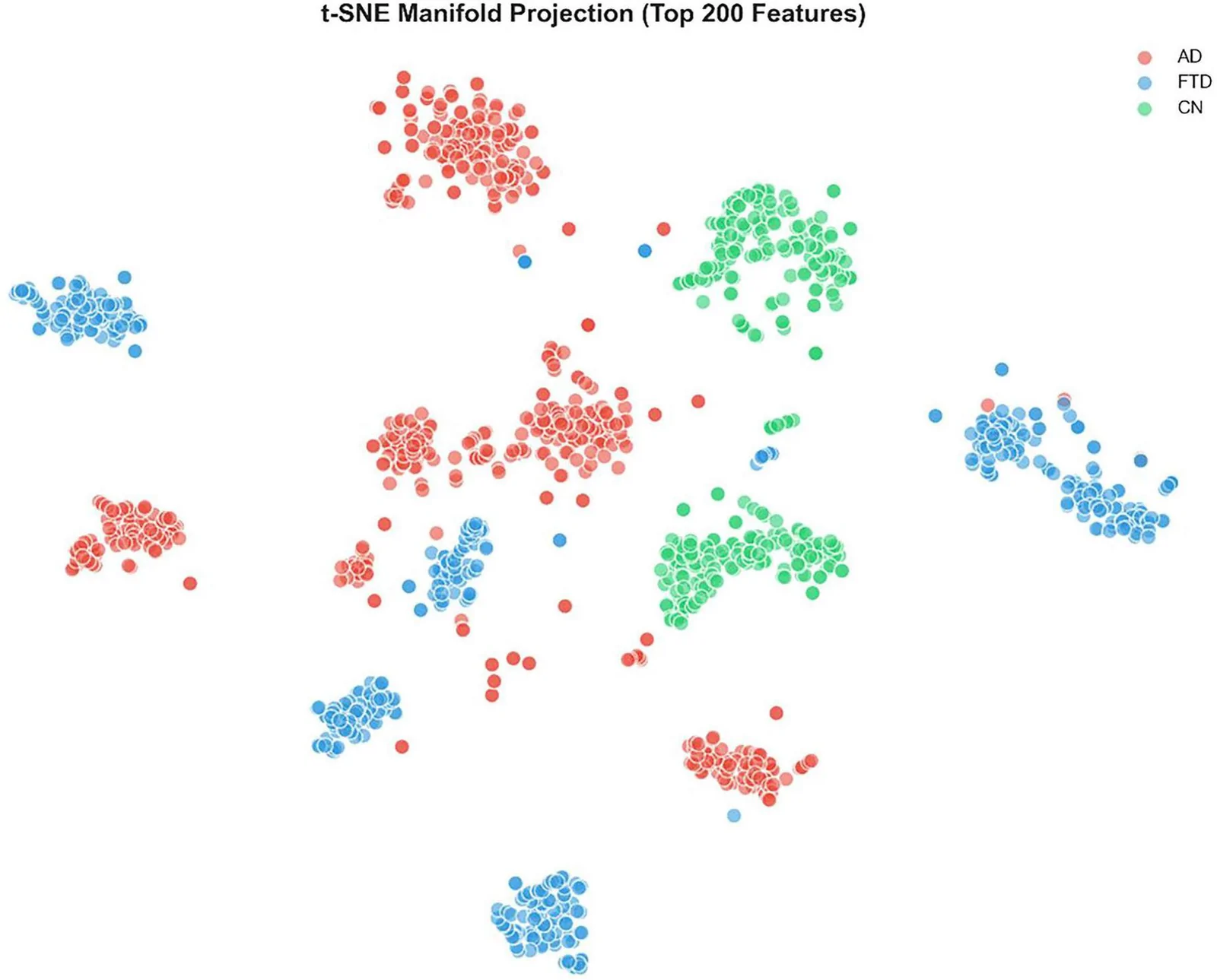
t-Distributed stochastic neighbor embedding (t-SNE) was applied to the optimized 200-dimensional feature space to visualize the intrinsic structure of the data. The resulting two-dimensional manifold projection revealed distinct clustering patterns corresponding to Alzheimer’s disease (AD), frontotemporal dementia (FTD), and cognitively normal (CN) groups. While t-SNE does not constitute a formal measure of class separability, the observed spatial organization suggests that the extracted reactivity and hemispheric asymmetric features capture diagnostically relevant differences among the groups prior to supervised classification.

Figure 4 shows the Subject-Level Confusion matrix whereas Figure 5 shows the t-distributed Neighbor Embedding.

#### 3.2.1 ROC curve and AUC analysis

Receiver Operating Characteristic (ROC) analysis demonstrates the robustness of the classifier across a spectrum of decision thresholds. The diagnostic performance is quantified by the Area Under the Curve (AUC), defined as:

- Frontotemporal dementia (FTD): AUC = 0.998
- Cognitive normal (CN): AUC = 0.995
- Alzheimer’s disease (AD): AUC = 0.993

**FIGURE 6:**
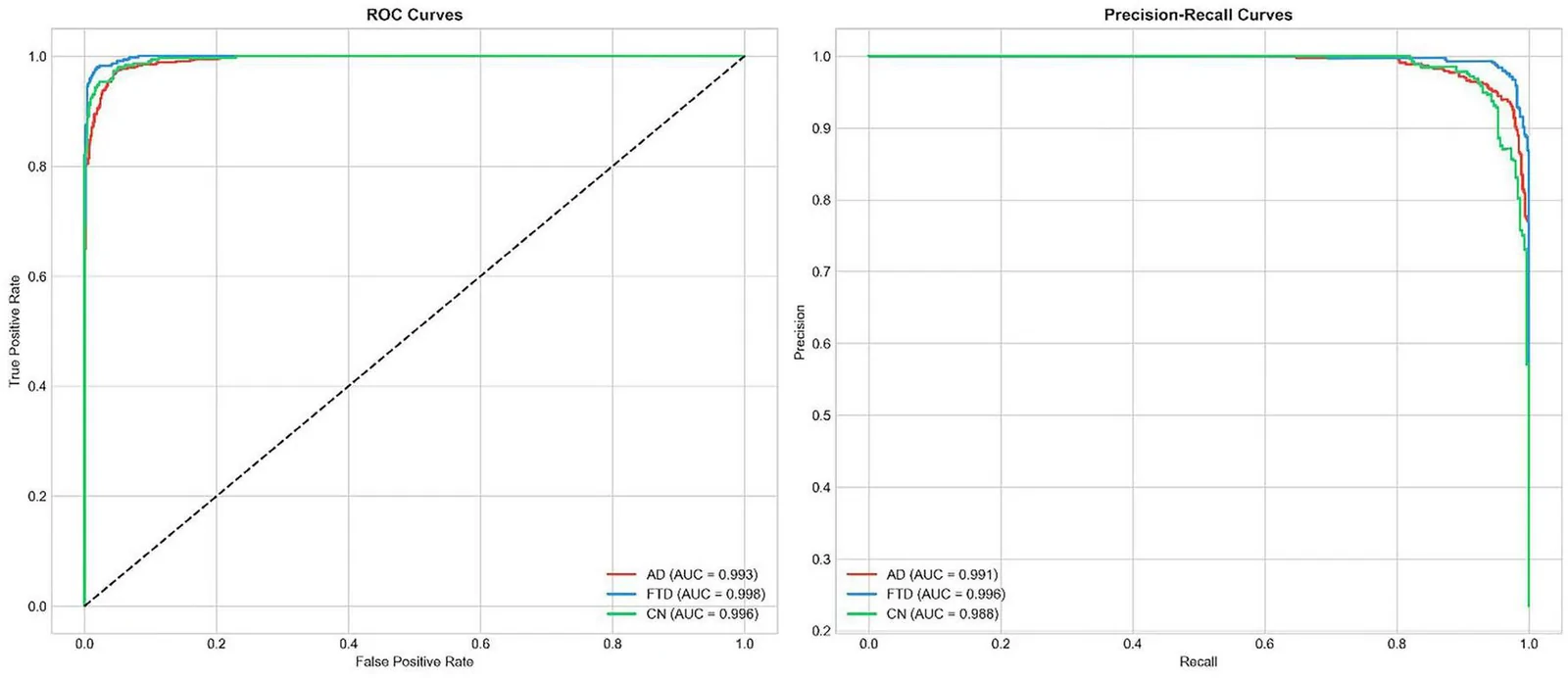
Receiver operating characteristic (ROC) and precision–recall (PR) curves. One-vs.-rest ROC curves depicting classifier discrimination performance across varying decision thresholds, with area under the curve (AUC) values ≥ 0.993 for all diagnostic classes. Precision–recall (PR) curves illustrating performance under class imbalance conditions, with sustained high precision observed for the minority FTD cohort, reaching a peak precision of 0.9907 at elevated recall levels.

Figure 6 shows the operating characteristic (ROC) and Precision–Recall (PR) Curves.

The high AUC ( > 0.99) across all classes suggests that the optimized 200-dimensional feature vector incorporating resting-state asymmetry and photic reactivity provides a stable representation of patterns associated with the pathologies (Santini et al., 2021).

### 3.3 Model stability and statistical reliability

The stability analysis yielded a mean segment-level cross-validation accuracy of 0.9846 ± 0.003 across the five internal training folds. Because each internal validation fold evaluated approximately 2,326 segments (20% of the 11,630 training segments), the reported standard deviation of 0.003 accurately reflects the tight variance in segment-level classification performance, confirming the model’s robustness against sampling variations prior to evaluation on the final holdout set. While the exact composition of the 200-feature subspace varied slightly across the five folds, core physiological markers such as Beta Asymmetry and Alpha Reactivity demonstrated extremely high selection stability, appearing 100% of the fold-wise subsets, supporting these features as primary biomarkers. Table 4 summarizes the matrices related to model stability and Statistical Reliability.

**TABLE 4:** Performance and interpretability metrics of the ensemble model for diagnostic classification and feature impact assessment.

Metric	Value/score	Scientific significance
Subject-independent five-fold stratified cross-validation	0.9846 ± 0.003	Measures model stability across diverse participant subsets.
Cohen’s kappa	0.9145	Validates that the accuracy significantly exceeds chance agreement (*p* < 0.001).
Standard deviation	0.003	Standard Deviation 0.003 Reflects fold-wise variability in segment-level accuracy across internal training folds.
Gain (max)	0.0160	Measures the maximum contribution of a single feature (Beta Asymmetry) to model certainty.

Statistical significance of the final classification performance was assessed using Cohen’s Kappa coefficient, which confirmed that agreement between predicted and true labels substantially exceeded chance-level expectation (section 2.8).

## 4 Discussion

The findings of this study indicate that a domain-guided, feature-optimized ensemble framework can yield multi-class classification of neurodegenerative pathologies, achieving a mean test accuracy of 95.63%. This performance suggests that electrophysiological approaches, when subjected to rigorous dimensionality reduction (RFE) may offer a computationally efficient and interpretable complement to deep learning approaches, particularly in data-constrained clinical environments. By constraining the feature space to the most discriminative dimensions, the model mitigates the Curse of Dimensionality (Bunterngchit et al., 2026; Liu and Gillies, 2016) often prevalent in small-cohort clinical studies, demonstrating that diagnostic precision appears associated with specific biological signals specifically hemispheric asymmetry and dynamic reactivity rather than the accumulation of high-dimensional noise.

### 4.1 Comparative analysis with existing literature

To contextualize the performance of the proposed framework, we compared our results against recent studies (2023–2026) utilizing comparable EEG datasets. The analysis, summarized in table, highlights the critical trade-off between model complexity and generalization capability. The proposed framework’s accuracy of 95.63% compares favorably with Deep Learning benchmarks, comparable to the current standard while utilizing significantly fewer computational parameters. Table 5 lists some Bench-marking comparisons of the classification methodologies.

**TABLE 5:** Benchmark comparison of classification methodologies, highlighting diagnostic accuracy and interpretability trade-offs between deep learning, connectivity-based models, and the proposed ensemble approach.

Study	Methodology	Task	Accuracy	Comparative observation
Ajra et al. (2023)	Shallow CNN	Multi-class	94.54%	Represents the current performance benchmark, achieving statistical parity with the proposed framework (Delta < 0.1%). However, while the deep learning architecture yields high accuracy, it offers limited transparency compared to the proposed ensemble, which explicitly isolates clinically interpretable biomarkers such as hemispheric asymmetry
Rostamikia et al. (2024)	SVM + connectivity	Multi-class	93.50%	A strong competitor using functional connectivity. Our ensemble outperformed this method by +0.95%, demonstrating that reactivity features may provide additional separation
Si et al. (2023)	Multilayer networks	AD vs. FTD	81.10%	Leveraged complex network topology features; however, the reliance on static measures without integration of dynamic reactivity metrics appears to constrain the model’s sensitivity, resulting in reduced diagnostic precision for the FTD class
Khan et al. (2025)	Hybrid TCN-LSTM	Multi-class	80.34%	Illustrates the challenges of applying complex temporal architectures (LSTM) to limited clinical cohorts. The performance differential suggests that it may offer stable performance and resistance to overfitting compared to deep learning models in data-constrained scenarios
Present study (2026)	Ensemble (XGB+RF)	Multi-class	95.63%	Optimization of the feature subspace yielded classification metrics comparative to deep learning benchmarks. These findings suggest that rigorous, parsimonious feature selection offers a strong alternative to high-complexity architectures, effectively reconciling the trade-off between predictive power and the biological interpretability required for clinical translation

### 4.2 Neurophysiological interpretability and formal logic

A defining attribute of the proposed framework is its interpretability. In contrast to opaque deep learning models, the decision-making mechanism of the ensemble was validated through SHAP (SHapley Additive exPlanations), a model-agnostic feature attribution method grounded in cooperative game-theoretic principles (Lundberg and Lee, 2018). SHAP quantifies the contribution of each feature by considering all possible coalitions of features and assigning credit to each feature’s marginal contribution to the prediction outcome. Formally, we operationalized the contribution ϕof feature *i*in the model prediction *f*(*x*) as (Equation 12):


$$\phi_{i}\,\left(f,x\right)=\underset{z^{{}^{\prime }}\subseteq x^{{}^{\prime }}}{\sum }\frac{|z^{{}^{\prime }}|!\left(M-|z^{{}^{\prime }}|-1\right)!}{M!}\,\left[f_{x}\,\left(z^{{}^{\prime }}\right)-f_{x}\,\left(z^{{}^{\prime }}\backslash i\right)\right]$$ (12)

where *f*_*x*_(*z*′) denotes the prediction of the model using feature subset *z*′, and *M*is the total number of features. This cooperative-game approach ensures that each feature’s influence is weighted by its contribution across all subsets, providing a principled basis for interpretability and establishing certain physiological drivers as candidate biomarkers. It should be noted that the SHAP attributions presented here were derived from a model refit on the full 88-subject cohort following cross-validation and therefore constitute exploratory biomarker signals rather than held-out validated findings. Their consistency with established neurophysiological literature lends biological plausibility, but prospective validation on an independent cohort is warranted before clinical attribution (Lundberg and Lee, 2018) The prominence of beta-band hemispheric asymmetry as the top-ranked SHAP feature is consistent with prior reports of asymmetric cortical disconnection in frontotemporal dementia, while the contribution of alpha-band reactivity aligns with established findings of attenuated photic-driving responses and reduced alpha reactivity in Alzheimer’s disease, reflecting impaired thalamocortical and visual pathway integrity (Perry and Hodges, 2000). From a practical clinical standpoint, the beta-band hemispheric asymmetry index consistently ranked as the most discriminative feature could serve as a computationally lightweight screening flag within a clinical decision-support system, alerting clinicians to atypical lateralization patterns warranting further neuropsychological evaluation. Similarly, attenuated alpha reactivity to photic stimulation may function as a complementary, quantifiable indicator of thalamocortical integrity, providing an objective correlate to supplement existing clinical assessments.

#### 4.2.1 Hemispheric asymmetry as a structural proxy

SHAP analysis identified Resting-state Beta Asymmetry (*rest_asym_ch7-9_beta*) as the highest-gain discriminative feature, corroborating recent findings in structural neuroimaging. Specifically, volumetric research by Pérez-Millan et al. (2025) introduced a “Cortical Asymmetry Index” (CAI) that demonstrates frontotemporal dementia (FTD) manifests with lateralized degeneration distinctly more than Alzheimer’s disease (AD) (Pérez-Millan et al., 2025). Mechanistically, by encoding the normalized power difference between homologous temporal channels (*T3-T4*), the model serves as an estimate of cortical lateralization. Distribution analysis indicates that AD subjects maintain near-zero variance (σ^2^0), whereas FTD subjects exhibit significantly elevated variance (*p* = 0.001), establishing asymmetry as a functional proxy of structural degeneration.

#### 4.2.2 Neural reactivity and dynamic plasticity

To address limitations inherent in static spectral features, we integrated a *Reactivity Vector* defined as ${\vec{V}}_{\text{DIFF}}=P_{\text{PHOTO}}-P_{\text{REST}}$, capturing the dynamic difference between photic stimulation and resting-state spectral power. Validation from Kang et al. (2025) shows early dementia is associated with stiffness in neural state transitions, measured by Leading Eigenvector Dynamics Analysis (LEiDA) (Kang et al., 2025). This is further supported by recent neurophysiological evidence demonstrating that functional connectivity and specific spectral bands, notably alpha power, are highly sensitive indicators of neural plasticity and cognitive modulation (Mahmood et al., 2024). The magnitude $\left|{\vec{V}}_{\text{DIFF}}\right|$thus reflects thalamocortical plasticity; functionally, this vector distinguishes FTD not only by *where* spectral power resides, but by *how* it fails to adapt under sensory stress, potentially reflecting dynamic network rigidity.

### 4.3 Computational rationale: addressing the Hughes phenomenon

The stability of the model evidenced by a low standard deviation in cross-validation (σ = 0.003) is attributable to rigorous management of the bias–variance trade-off, particularly in optimizing feature dimensionality relative to sample size.

#### 4.3.1 Curse of dimensionality and feature optimization

In its initial high-dimensional state (*p* = 1,014) with a fixed sample size of *n* = 88, the model was susceptible to the *Hughes phenomenon*, a manifestation of the *curse of dimensionality* in machine learning whereby classifier performance improves with increasing features only up to a point and then deteriorates as data become sparse and overfitting increases (Zollanvari, 2016). To mitigate this effect and enhance generalizability, we applied Recursive Feature Elimination (RFE) to reduce the feature space to *p* = 200, thereby improving the effective ratio of signal density to feature volume and lowering estimation error. To strictly prevent information leakage, the RFE protocol was embedded within the cross-validation loop. For every fold, RFE was fitted using only the training subjects to select an independent set of 200 features, which was then evaluated on the held-out test subjects. While the exact composition of the 200-feature subspace varied slightly across the five folds, core physiological markers such as Beta Asymmetry and Alpha Reactivity demonstrated extremely high selection stability, appearing 100% of the fold-wise subsets. The final, universal 200-feature subspace and the global SHAP attributions presented in section 4.2 were derived by refitting this validated pipeline on the complete dataset, representing the final clinical model. The overall expected prediction error can be decomposed as (Equation 13):


$$E\,\left[{\left(\hat{f}\,\left(x\right)-f\,\left(x\right)\right)}^{2}\right]=\text{Bias}\,{\left[\hat{f}\,\left(x\right)\right]}^{2}+\text{Var}\,\left[\hat{f}\,\left(x\right)\right]+\sigma_{\text{noise}}^{2}$$ (13)

By pruning non-informative dimensions that contributed primarily to noise fitting, RFE reduced the *variance* term ($\text{Var}\,\left[\hat{f}\,\left(x\right)\right]$), while domain-specific features such as asymmetry and reactivity maintained low bias (Hughes, 1968). It is acknowledged that, despite the use of subject-independent cross-validation to mitigate overfitting, the overall cohort size of 88 participants constrains the statistical power and generalizability of the reported findings. Performance metrics reported here, should be interpreted as estimates derived from a single-site, modestly sized cohort; replication on larger, multi-site, and demographically diverse populations is necessary before these biomarkers can be considered for clinical translation.

#### 4.3.2 Ensemble integration strategy

The final classification probability *P*_*final*_ was derived via a weighted soft-voting fusion of Extreme Gradient Boosting (XGBoost) and Random Forest (RF) classifiers (Equation 14):


$$P_{\text{final}}=\alpha \cdot P_{\text{XGB}}+\left(1-\alpha \right)\cdot P_{\text{RF}}$$ (14)

where α = 0.6. XGBoost functions as a low-bias learner, minimizing a cost-sensitive loss function (Equation 15):


$$L\,\left(\theta \right)=-w_{c}\,y_{i}\,\text{log}\,\Sigma \,\left({\hat{y}}_{i}\right)$$ (15)

with class weight *w*_*c*_ = 2.0 for the minority FTD class to prioritize gradient updates toward reducing false negatives. The RF component (with weight 1−α = 0.4) stabilizes variance via bootstrap aggregation (bagging), decorrelating individual trees and smoothing the final decision boundary, enhancing resistance to outliers in the reduced feature space (Karlos et al., 2020).

### 4.4 Clinical implications and limitations

Ethically, the framework conforms to the principle of *primum non nocere (first do not harm)*. By optimizing the cost function toward the minority class, the model achieved an FTD precision of 0.9907, associated with reduced risk of false-positive diagnoses as an important consideration given the severe psychological and medical consequences of misdiagnosing treatable conditions or mistaking healthy aging for terminal dementia (White and Algeri, 2023).

While the ensemble framework achieves high diagnostic precision, several methodological considerations remain. The current reliance on a single-center cohort necessitates further validation on diverse, multi-center datasets to account for clinical variability in hardware and demographics. The proposed framework’s reliance on spectral and statistical features derived from standard EEG frequency bands positions it as hardware-agnostic in principle; however, inter-site variability in electrode impedance, amplifier gain, and acquisition sampling rates may influence feature distributions across centers. Future deployment should incorporate site-harmonization strategies such as z-score normalization per site or transfer learning to preserve diagnostic consistency across heterogeneous clinical environments . Furthermore, the cross-sectional nature of this study limits insights into longitudinal biomarker stability and disease progression.

Future research will focus on the following high-priority areas:

- Multimodal Integration: Fusing EEG-derived reactivity and asymmetry markers with structural MRI indices, such as the Cortical Asymmetry Index (CAI), to develop a more holistic diagnostic profile.
- External Validation: Evaluating cross-site strength by testing the framework on independent cohorts with varying acquisition protocols.
- Prognostic Modeling: Utilizing longitudinal data to track the evolution of neural stiffness and lateralized atrophy, potentially enabling early prognostic screening.
- Cohort Expansion: Scaling the sample size to a larger, statistically powered cohort to mitigate the risk of small-sample variance and verify that the observed high-precision classification metrics are robust rather than artifacts of a limited participant pool.

Ultimately, prospective clinical validation will be required to transition this framework from a strong computational model into a routine diagnostic workflow.

## 5 Conclusion

This study presented a domain-guided heterogeneous ensemble framework for the automated differential diagnosis of Alzheimer’s Disease (AD) and Frontotemporal Dementia (FTD) using scalp EEG. The proposed model achieved a multi-class accuracy of 95.63%, with a high FTD-specific precision of 0.9907, while remaining computationally efficient and biologically interpretable. By operationalizing the Reactivity Hypothesis through the integration of photic stimulation (*V*_*diff*_) and the Hemispheric Asymmetry Index (HAI), the model was able to capture the dynamic loss of neural plasticity and lateralized atrophy often missed by static resting-state protocols. Compared with deep learning approaches reported in recent literature, the present framework demonstrates competitive diagnostic performance while maintaining computational efficiency and interpretability. Although complex architectures like Hybrid TCN-LSTMs or Transformer-based models may achieve marginally higher raw accuracy through extensive parameterization, their high computational demands and lack of transparency may limit clinical interpretability (Bunterngchit et al., 2024; Bunterngchit et al., 2025; Bunterngchit et al., 2026). In practical terms, the XGBoost/Random Forest ensemble requires no GPU infrastructure for either training or inference, however in this case with use of GPU, full model training completing in under 7 min. The results demonstrate that carefully engineered, feature-optimized ensembles can offer a practical balance between high diagnostic performance and clinical explainability. By reducing the feature space to a parsimonious 200 dimensions, this framework functions effectively on standard consumer-grade hardware, making it potentially suitable for deployment in resource-constrained clinical environments where neuroimaging is inaccessible. In summary, the findings indicate that dynamic, interpretability-first machine learning models hold strong potential for help address the specificity gap in dementia subtyping, proposing a scalable and non-invasive alternative to complex deep learning architectures.

## Data Availability

The datasets analyzed in this study are publicly available on OpenNeuro. The resting-state EEG dataset can be accessed at https://openneuro.org/datasets/ds004504 and the photic stimulation dataset at https://openneuro.org/datasets/ds006036.
